# The Role of Glucose Metabolism on Porcine Oocyte Cytoplasmic Maturation and Its Possible Mechanisms

**DOI:** 10.1371/journal.pone.0168329

**Published:** 2016-12-20

**Authors:** Bao Yuan, Shuang Liang, Jeong-Woo Kwon, Yong-Xun Jin, Shun-Ha Park, Hai-Yang Wang, Tian-Yi Sun, Jia-Bao Zhang, Nam-Hyung Kim

**Affiliations:** 1 Department of Laboratory Animals, College of Animal Sciences, Jilin University, Changchun, Jilin, China; 2 Molecular Embryology Laboratory, Department of Animal Sciences, Chungbuk National University, Cheongju, Chungbuk, South Korea; Institute of Zoology Chinese Academy of Sciences, CHINA

## Abstract

In the present study, we investigated the potential role of glucose and pyruvate in the cytoplasmic maturation of porcine oocytes by investigating the effect of glucose and/or pyruvate supplementation, in the presence or absence of 10% porcine follicular fluid (PFF), on meiotic maturation and subsequent embryo development. In the absence of 10% PFF, without exogenous addition of glucose and pyruvate, the medium seemed unable to support maturation. In the presence of 10% PFF, the addition of 5.6 mM glucose and/or 2 mM pyruvate during in vitro maturation of cumulus enclosed oocytes increased MII oocyte and blastocyst rates. In contrast, oocytes denuded of cumulus cells were not able to take full advantage of the glucose in the medium, as only pyruvate was able to increase the MII rate and the subsequent early embryo developmental ability. Treatment of cumulus enclosed oocytes undergoing maturation with 200 μM dehydroepiandrosterone (DHEA), a pentose phosphate pathway inhibitor, or 2 μM iodoacetate (IA), a glycolysis inhibitor, significantly reduced GHS, intra-oocyte ATP, maternal gene expression, and MPF activity levels. DHEA was also able to increase ROS and reduce the levels of NADPH. Moreover, blastocysts of the DHEA- or IA-treated groups presented higher apoptosis rates and markedly lower cell proliferation cell rates than those of the non-treated group. In conclusion, our results suggest that oocytes maturing in the presence of 10% PFF can make full use of energy sources through glucose metabolism only when they are accompanied by cumulus cells, and that pentose phosphate pathway (PPP) and glycolysis promote porcine oocyte cytoplasmic maturation by supplying energy, regulating maternal gene expression, and controlling MPF activity.

## Introduction

In a variety of mammals, glucose metabolism and pyruvate play an important role in oocyte maturation and subsequent early embryonic development [1–3]. In an in vitro oocyte culture system, a decrease in glucose concentration was shown to significantly inhibit meiotic divisions, subcapsular maturation, and subsequent embryonic development in bubaline and bovine oocytes[4, 5]. Moreover, the addition of a sufficient amount of glucose to the IVM medium significantly enhances IVM and subsequent developmental capacity of bovine and porcine oocytes [6–8].

Mammalian oocytes metabolize glucose primarily through glycolysis, PPP, and the tricarboxylic acid cycle (TAC) [1, 9]. PPP and/or glycolysis appear to play a key role in the resumption of meiosis in mouse oocytes [1, 10]. Gonadotropin reportedly promotes mouse oocytes to interact with cumulus cells via the interstitial space [11], but only when sufficient glucose is present in the IVM medium [12]. Studies have shown that there is a correlation between resumption of meiosis and increased glycolysis and PPP [10, 12]. However, high concentrations of glucose in the IVM medium can inhibit oocyte maturation [9].

Although several researchers have reported effects of glucose metabolism on oocyte maturation, knowledge of the underlying mechanism(s) is still limited [8, 13]. Moreover, even though other studies have observed that glucose metabolism during oocyte maturation can significantly affect in vitro fertilization and subsequent post-embryonic development [11, 14–17], the effect of glucose metabolism on oocyte cytoplasm maturation has not been studied so far.

Studies on IVM of porcine oocytes, the use of chemical constituents in culture, and the application of glycolysis and PPP inhibitors, have shown that glucose metabolism can influence the maturation of porcine oocytes [2]. The effects of PPP and/or glycolysis on in vitro nuclear maturation of porcine oocytes have also been demonstrated [2]. The studies of Sturmey et al. showed that porcine oocytes undergo energy depletion during maturation, suggesting that a high level of glucose must be maintained in the medium during IVM in order for sufficient pyruvate to be obtained, which in turn interacts with oxaloacetate to fuel TAC [18]. However, the mechanism through which glucose and pyruvate metabolism affect the maturation of oocyte cytoplasm is not fully understood.

The objective of the present research was to evaluate the influence of glucose metabolism on porcine oocyte cytoplasmic maturation, which could expand our knowledge on the mechanisms of glucose metabolism in oocytes maturation. In this research, we investigated the MII oocytes with respect to reactive oxygen species (ROS) levels, glutathione (GSH) levels, intra-oocyte ATP and NADPH levels, maturation-promoting factor (MPF) activity, and early embryonic development (e.g., apoptotic and proliferation rates in resultant blastocysts).

## Materials and Methods

### Ethics statement

This study was carried out in strict accordance with the recommendations in the Guide for the Care and Use of Laboratory Animals of Jilin University. The protocol was approved by the Institutional Animal Care and Use Committee of Jilin University (No. 20160303, Permit Number: 3120012).

### Reagents

Unless otherwise stated, all reagents were purchased from Sigma-Aldrich (St. Louis, MO, USA).

### Collection and IVM of porcine oocytes

The ovaries of pigs were retrieved from the slaughterhouse within 2 h from death. COCs were extracted from the 3–6 mm diameter follicles of the porcine ovaries and washed three times with Tyrode's Lactate HEPES (TL-HEPES). The collected COCs were maturated in IVM medium for 44 h at 38.5°C in a humidified atmosphere of 5% CO_2_/95% air. The IVM medium was based on the modified North Carolina State University 37 medium (NCSU37) [16] and contained 108.73 mM NaCl, 25.07 mM NaHCO_3_, 4.78 mM KCl, 1.19 mM KH_2_PO_4_, 1.19 mM MgSO_4_, 1.70 mM CaCl_2_, and 1.00 mM Glutamine. This medium was stored at 4°C and used within 2 weeks from preparation. Before use, the medium was supplemented with 0.6 mM L-cysteine, 10 ng/mL epidermal growth factor, 10 IU/mL luteinizing hormone (LH), 10 IU/mL follicle-stimulating hormone (FSH), 5 mg/mL insulin, and 10% v/v porcine follicular fluid (PFF). IVM medium not supplemented with PFF was used in certain experiments.

According to previous studies [1, 2], various concentrations of glucose (0, 0.56, or 5.6 mM) or pyruvate (0, 1, or 2 mM) were added to the IVM medium. After 42–44 h of IVM, the COCs were washed with TL-HEPES supplemented with 1 mg/mL hyaluronidase and 0.1% PVA to remove cumulus cells. Oocytes that had discharged the first polar body were picked for further experiments.

### Parthenogenetic activation and in vitro culture of pig oocytes

In accordance with the activation protocol used in our previous experiments, MII stage oocytes were selected[19]. Denuded oocytes with homogeneous cytoplasms were selected, gradually equilibrated in activation solution, and subjected to a 1.0-kV/cm electric pulse for 60 μs. Activated oocytes were incubated in PZM-5 medium with 2 mM cytochalasin B for 3 h. Next, 40–50 post-activation oocytes were cultured in PZM-5 for another 7 days, and the resultant embryo cultures were maintained at 38.5°C in 5% CO_2_.

### Measurement of ROS and GSH levels in MII oocytes

In order to determine the role of glucose metabolic pathway, MII stage oocytes were transferred in medium supplemented with DHEA or IA. To detect the levels of ROS, the oocytes were incubated with 10 μM 2′,7′-dichlorodihydrofluorescein diacetate (H2DCFDA; Thermo Fisher Scientific, Waltham, USA) for 15 min, followed by spectroscopy (green fluorescence, UV filters, 490 nm). To detect the levels of GSH, the oocytes were incubated with 10 μM 4-chloromethyl-6,8-difluoro-7-hydroxycoumarin (CMF_2_HC) Cell Tracker Blue dye (Thermo Fisher Scientific) for 15 min, followed by spectroscopy (blue fluorescence, UV filters, 370 nm). The oocytes of the different groups were identically processed with respect to incubation, washing, installation, and imaging. Image J software (Public Domain) was used to analyze the fluorescence intensity of oocytes. Three independent experiments were performed.

### Determination of intra-oocyte ATP levels

ATP levels in oocytes were measured using the ATP Determination Kit (Thermo Fisher Scientific) according to the kit instructions. Fifty cumulus-free oocytes were disrupted by sonication. After centrifugation (8,000 × *g*, 5 min), 10 μL supernatant samples were collected, and the amount of ATP in each sample (by extension the amount per oocyte) was determined using an ATP standard curve.

### Determination of intra-oocyte NADPH

The NADPH content of the oocytes was measured using an NADPH assay kit (Nanjing Jiancheng Bioengineering Institute, Jiangsu, China), according to the kit instructions. Briefly, 100 μL of the alkaline extract containing 100 oocyte-free oocytes was transferred to a 1.5-mL centrifuge tube. After sonication and centrifugation, the supernatant was mixed and neutralized with the same volume of acid extract. The amount of NADPH in each sample was divided by the number of oocytes to obtain the intracellular NADPH concentration of each oocyte.

### MPF activity assay in MII oocytes

The Cdc2/Cdk1 Kinase Assay Kit (MBL, Nagoya, Japan) was used to quantify p34^cdc2^ kinase activity [20, 21]. Briefly, thirty oocytes were washed 3 times with sample buffer. Oocyte extract (5 μL) was mixed with kinase assay buffer (45 μL). The mixture was placed in an incubator at 30°C for 30 min. The reaction was terminated by adding 200 μL of 50 mM ethylene glycol tetraacetic acid (EGTA). The OD was measured at 492 nm. Three independent experiments were performed.

### Real-time reverse transcription PCR

Total RNA extraction and cDNA synthesis were performed as described previously [22]. Briefly, 50 MII-stage oocytes or 20 blastocysts were used to extract mRNA using the Ambion Dynabeads mRNA Direct Kit (Thermo Fisher Scientific). Isolated mRNA was reverse-transcribed with the SuperScript Reverse Transcriptase Kit (LeGene Biosciences, San Diego, CA, USA) using oligo(dT)12–18 primers, followed by real-time PCR in a Bio-Rad CFX PCR cycler (Hercules, CA, USA) with the primers that were described in our previous paper [19]. Gene expression was analyzed with the 2^-ΔΔCt^ method [23], using glyceraldehyde 3-phosphate dehydrogenase (*GAPDH*) as a normalizer gene. Three separate experiments were performed, each containing three samples.

### Confocal and counting the number of nuclei per blastocyst

We performed the procedures as previously described [19]. Briefly, blastocysts were fixed for 30 minutes in 3.7% paraformaldehyde prepared in phosphate buffered saline with 0.1% (w/v) polyvinyl alcohol (PBS-PVA). Afterwards, they were washed with PBS, and then transferred to 0.3% Triton X-100 for 1 h. Thereafter, they were washed twice with PBS, and incubated in the dark for 1h after the addition of fluorescein-conjugated dUTP and terminal deoxynucleotidyl transferase. After a 1 h incubation with 10 μg/mL Hoechst 33342 and 50 mg/mL RNase A, the blastocysts were examined using a laser scanning confocal microscope. The total number of cells and the number of apoptotic cells were determined using ImageJ software (Public Domain).

### 5-Bromo-deoxyuridine (BrdU) analysis

The BrdU assay was performed as previously described [24] with minor modifications. Briefly, blastocysts were incubated in 100 mM BrdU with 5% CO_2_ at 39°C for 6 h. Blastocysts were washed with PBS containing Tween 20 (PBST), fixed in ice-cold methanol for 20 min, washed again, and then permeabilized at room temperature(20–25°C) for 2 min with 0.1% Triton X-100 (prepared in PBS-PVA). Blastocysts were then washed and treated with 2 N HCl at room temperature for 30 minutes. After washing, they were incubated with mouse anti-BrdU monoclonal antibody at a dilution of 1:10 overnight at 4°C. Incubation with the secondary antibody (rabbit anti-mouse immunoglobulin G-FITC polyclonal antibody, 1:500 dilution) was performed at room temperature for 1 h. Finally, the blastocysts were stained with 10 μg/mL Hoechst 33342, fixed on glass slides, and observed under a confocal laser scanning microscope. The number of proliferating cells was determined using ImageJ.

### Statistical analyses

All experimental results were analyzed using IBM SPSS Statistics v19 (IBM, Armonk, NY, USA). One-way ANOVA and Chi-square test were performed to evaluate the statistical significance of differences, all percentage data were subjected to arc sine transformation before statistical analysis. A p-value lower than 0.05 was considered statistically significant.

## Results

### Effects of glucose and pyruvate on IVM of porcine oocytes

**Fig 1:**
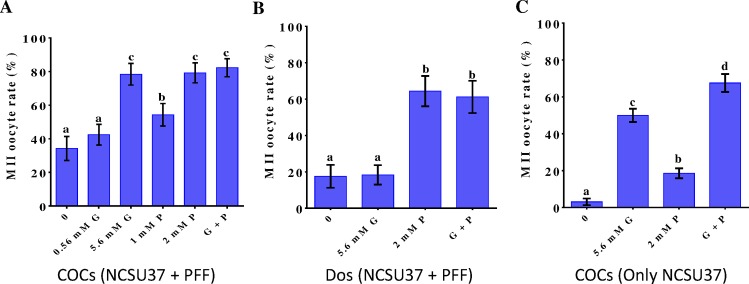
Effect of adding various concentrations of glucose or pyruvate on IVM of porcine oocytes. (A) COCs were cultivated in NCSU37 with 10% PFF and different concentrations of glucose or pyruvate. (B) DOs were cultivated in NCSU37 with 10% PFF and supplemented with glucose and/or pyruvate.(C) COCs were cultivated in NCSU37 with glucose and/or pyruvate. Control: no exogenous glucose/pyruvate; G: glucose-only supplementation; P: pyruvate-only supplementation; G + P: supplementation with both glucose and pyruvate. Differences between bars superscripted with different letters (a, b, c, or d within same graph)were statistically significant (p < 0.05). Values are the mean ± standard deviation from three independent experiments.

To evaluate the effect of glucose and pyruvate on porcine oocyte maturation, COCs or DOs were maturated in the presence of different concentrations of glucose, pyruvate, or both. In the case of COCs, maturation in NCSU37 with 10% PFF resulted in 34.21 ± 7.13% of oocytes reaching the MII stage. As seen in Fig 1A, supplementation with 5.6 mM glucose, 2mM pyruvate, or both, resulted in similar increases in the rates of nuclear maturation (78.39 ± 6.46, 79.23 ± 5.95, and 82.27 ± 5.34, respectively; p < 0.05 vs. controls). On the other hand, maturation of DOs in NCSU37 with 10% PFF led to a MII-stage rate of 17.57 ± 6.27%. As seen in Fig 1B, supplementation with 5.6 mM glucose did not lead to a significant increase in MII oocyte rate (18.35 ± 5.32%; p > 0.05 vs. controls), probably because PPF can by itself cover the energy needs of the nuclear maturation process. In contrast, supplementation with 2 mM pyruvate, alone or in combination with 5.6 mM glucose, increased nuclear maturation rates to 64.39 ± 8.33% and 61.23 ± 8.85%, respectively (p < 0.05 vs. controls and cells supplemented only with 5.6 mM glucose) (Fig 1B). We also examined the maturation of COCs in NCSU37 medium without PFF. Without exogenous addition of glucose and/or pyruvate, the medium seemed unable to support maturation, as the rate of MII oocytes was only 3.05 ± 1.8%. When the medium was supplemented with 5.6 mM glucose, 2mM pyruvate, or both, the rates were significantly increased to 49.96 ± 3.58%, 18.54 ± 2.73%, and 67.53 ± 4.89%, respectively (p < 0.05 vs. controls) (Fig 1C).

### Effects of glucose and/or pyruvate supplementation during IVM on subsequent early embryo development

**Fig 2:**
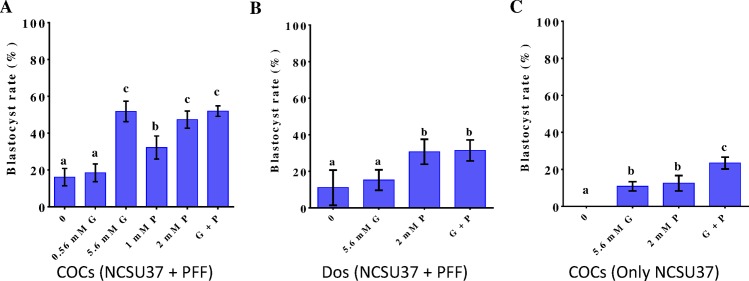
Effect of various concentrations of glucose or pyruvate during IVM on porcine oocyte development to the blastocyst stage. (A) Blastocyst rates when COCs were cultivated in NCSU37 with 10% PFF, without or with supplementation with different concentrations of glucose and/or pyruvate. (B) Blastocyst rates after DOs were cultivated in NCSU37 with 10% PFF supplemented with glucose, pyruvate, or both. (C) Blastocyst rates when COCs were cultivated in NCSU37 with glucose and/or pyruvate (P). Control: no exogenous glucose/pyruvate; G: glucose-only supplementation; P: pyruvate-only supplementation; G + P: supplementation with both glucose and pyruvate. Differences between bars superscripted with different letters (a, b, or c within same graph) were statistically significant (p < 0.05). Values are the mean ± standard deviation from three independent experiments.

To determine the effects of glucose and/or pyruvate supplementation during IVM on the subsequent embryonic development, we examined the development of activated porcine oocytes after maturation in NCSU37 with 10% PFF, in the absence (controls) or presence of glucose and/or pyruvate. In the case of COCs (Fig 2A), addition of 5.6 mM glucose, 2 mM pyruvate, or both, resulted in similar increases in blastocyst rates (51.71 ± 5.54, 47.26 ± 4.67, and 51.93 ± 2.84, respectively; p < 0.05 vs. controls). In the case of DOs maturing in NCSU37 with 10% PFF (Fig 2B), the addition of 5.6 mM glucose alone led to a rate (15.25 ± 5.59%) similar to that of controls, while supplementation with 2 mM pyruvate, alone or in combination with 5.6 mM glucose, significantly increased blastocyst rates (30.75 ± 6.83% and 31.44 ± 5.74%, respectively; p < 0.05 vs. controls and cells treated only with 5.6 mM glucose). We repeated the experiment using COCs maturing in NCSU37 medium without PFF. Without addition of exogenous glucose or pyruvate, the medium was unable to support the succeeding embryonic development (Fig 2C). Adding 5.6 mM glucose or 2 mM pyruvate significantly increased blastocyst rates (10.82 ± 2.51% and 12.50 ± 4.17%, respectively; p < 0.05 vs. controls), while addition of both pyruvate and glucose raised levels even higher (23.40 ± 3.19%; p < 0.05 vs. control, glucose-only, and pyruvate-only samples).

These results suggest that PFF is important to oocyte maturation and subsequent embryo development. Moreover, supplementation with glucose alone was only effective in COCs and not in DOs, indicating that cumulus cells are necessary for exogenous glucose to exert its effect on maturation and early embryonic development of oocytes.

### Effects of DHEA and IA in IVM and subsequent embryo development of porcine oocytes

**Fig 3:**
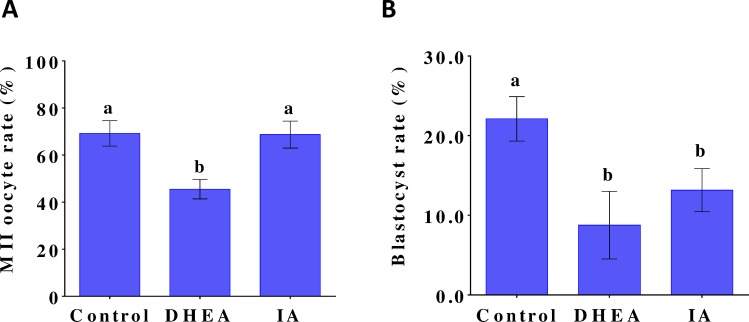
Effects of DHEA and IA treatment during IVM of COCs on oocyte maturation and embryo development. (A) MII oocyte rates and (B) blastocyst rates after maturation of COCs in NCSU37 supplemented with 10% PFF, 5.6 mM glucose, and 2 mM pyruvate, in the absence (controls) or presence of 200 μM DHEA or 2 μM IA. Values are the mean ± standard deviation from three independent experiments. Differences between bars superscripted with different letters (a or b within same graph) were statistically significant (p < 0.05).

COCs were maturated in NCSU37 supplemented with 10% PFF, 5.6 mM glucose, and 2 mM pyruvate, in the absence (controls) or presence of 200 μM DHEA (a PPP inhibitor) or 2μM IA (a glycolysis inhibitor). As seen in Fig 3A, DHEA significantly (p < 0.05) decreased the oocyte nuclear maturation rate from 69.25 ± 5.40% (controls) to 45.52 ± 4.14%, whereas IA did not have a significant effect on the rate of MII oocytes. With respect to the succeeding embryo development, the presence of 200 μM DHEA or 2 μM IA significantly (p < 0.05) reduced the blastocyst rate from 22.11 ± 2.79% (controls) to 8.76 ± 4.25% and 13.15 ± 2.70%, respectively (Fig 3B).

### Effect of DHEA or IA on Intracellular Levels of ROS and GSH

**Fig 4:**
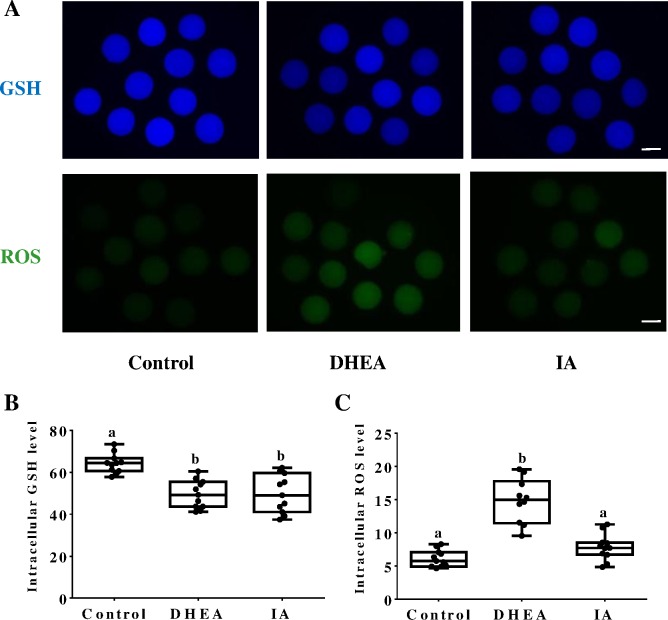
The effects of DHEA and IA on GSH and ROS levels in MII oocytes. (A) MII oocytes maturated in control IVM medium or medium supplemented with DHEA or IA were dyed with CMF_2_HC (Cell Tracker Blue) and H_2_DCFDA to detect GSH and ROS levels, respectively. Scale bar indicates 100 μm. (B) Effect of DHEA or IA supplementation during IVM on intracellular GSH levels in mature oocytes. (C) Effect of DHEA or IA supplementation during IVM on intracellular ROS levels in mature oocytes. Differences between bars superscripted with different letters (a or b within same graph) were statistically significant (p <0.05). The experiment was repeated three times.

To determine the mechanisms through which DHEA and IA influence porcine oocyte maturation, the levels of GSH and ROS were determined in the corresponding post-IVM oocytes. The levels of GSH were significantly lower (p < 0.05) in both DHEA- (49.50 ± 6.70 pixels/oocyte) and IA-treated oocytes (49.72 ± 9.01 pixels/oocyte) than in the control group (64.22 ± 4.61 pixels/oocyte) (Fig 4A and 4B). With respect to ROS, its levels were significantly (p < 0.05) higher in the DHEA-treated oocytes (14.81 ± 3.34 pixels/oocyte) than in the control group (6.11 ± 1.27 pixels/oocyte) and in the IA-treated oocytes (7.82 ± 1.97pixels/oocyte) (Fig 4A and 4C).

### Effects of DHEA or IA on intra-oocyte ATP and NADPH levels during oocyte maturation

**Fig 5:**
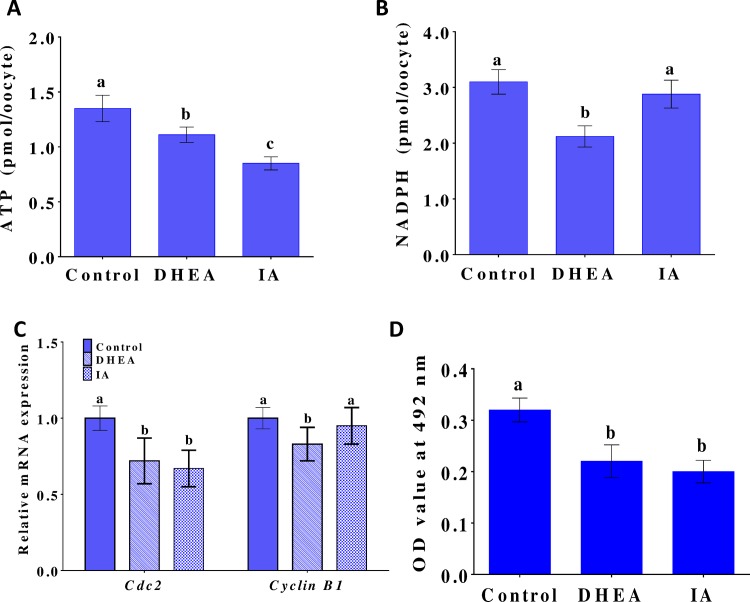
Intra-oocyte ATP and NADPH levels and MPF activity in MII oocytes. (A) Intra-oocyte ATP levels, (B) Intra-oocyte NADPH levels, (C) Expression levels of the maternal transcripts *cdc2* and *cyclin B1*, and (D) MPF activity in MII oocytes, after porcine COC maturation in NCSU37 with 10% PFF, 5.6 mM glucose, and 2 mM pyruvate, in the absence (controls) or presence of 200 μM DHEA or 2 μM IA. Each treatment was repeated three times. In each experiment, samples contained 50 (for ATP, mRNA, and MPF activity levels) or 100 (for NADPH levels) cumulus-free oocytes. Differences between bars superscripted with different letters (a, b, or c within same graph) were statistically significant (p < 0.05).

Intra-oocyte levels of ATP and NADPH were measured after maturation of COCs in NCSU37 supplemented with 10% PFF, 5.6 mM glucose, and 2 mM pyruvate, in the absence (controls) or presence of 200 μM DHEA or 2 μM IA. Treatment with DHEA or IA significantly reduced intra-oocyte ATP levels (Fig 5A), suggesting that both PPP and glycolysis produce ATP during oocyte maturation. Treatment with DHEA significantly (p < 0.05) reduced the level of NADPH, while IA only had a mild effect (Fig 5B). This result suggests that PPP plays a more important role in maintaining oocyte redox potential compared to glycolysis.

### The effects of DHEA or IA on maternal gene expression and MPF activity

Regulation of maternal gene expression is an important biological process in oocyte maturation and early embryo development. We examined the effects of DHEA and IA on this process by investigating the expression of two representative maternal transcripts, *cdc2* and *cyclinb1*, which encode the regulatory subunits of MPF. The mRNA levels were measured in MII oocytes after IVM of COCs in the presence of DHEA or IA. As seen in Fig 5C, both DHEA and IA significantly (p < 0.05) reduced *cdc2* mRNA levels compared to non-treated controls, while DHEA also reduced *cyclinb1* mRNA levels (p < 0.05 vs. controls) (Fig 5C). We also measured the activity of p34^cdc2a^, which is the protein encoded by the *cdc2* gene. MII oocytes that had been treated with DHEA or IA during IVM displayed significantly (p < 0.05) reduced p34^cdc2a^ activity compared with the control group (Fig 5D).

### The effects of DHEA and IA on porcine blastocyst apoptosis

**Fig 6:**
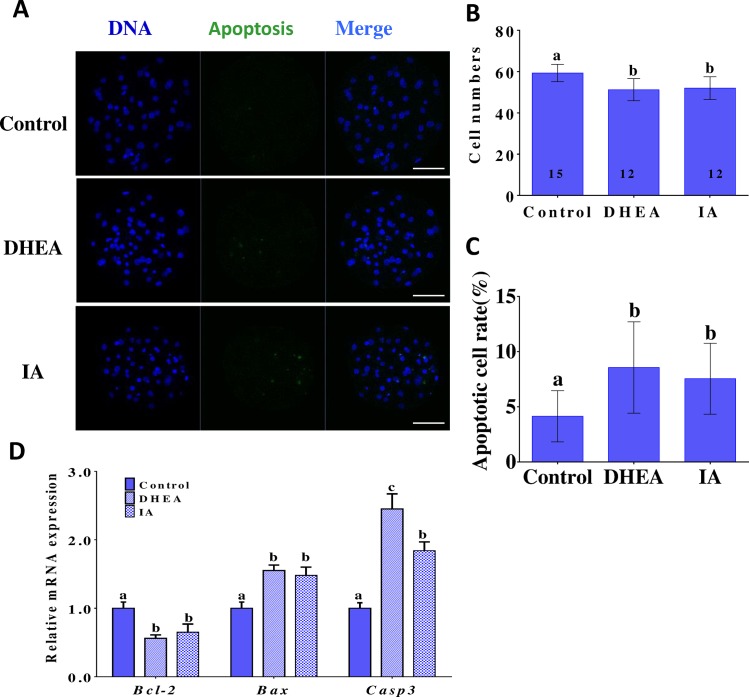
Typical confocal images showing nuclear DNA and apoptotic cells in 7 d porcine blastocysts. (A) Immunofluorescent staining of apoptotic cells in blastocysts from control, DHEA-treated, and IA-treated groups (×400). Scale bar indicates 100 μm. (B) The total number of cells per blastocyst in different groups. (C) Apoptotic cell rate per blastocyst in different groups. The number of observed oocytes in each experimental group is displayed inside the bars. (D) *Bcl-2*, *Bax*, and *Casp3* mRNA levels in 7 d porcine blastocysts from controls and groups treated with DHEA or IA during IVM. Values are the mean ± standard deviation of the mean. Differences between bars superscripted with different letters (a, b, or c within same graph) were statistically significant (p < 0.05).

**Fig 7:**
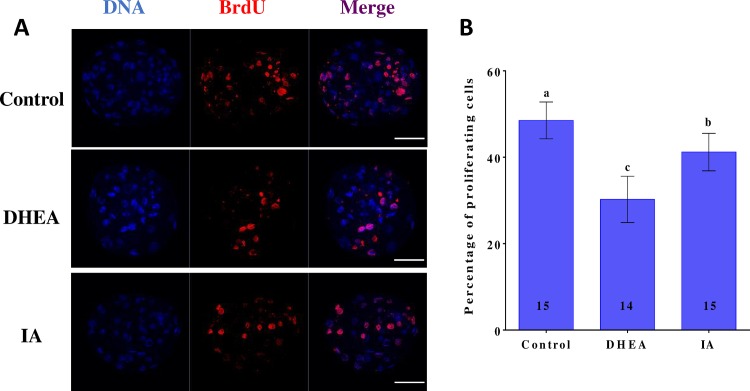
Typical confocal images showing nuclear DNA and proliferating cells in 7 d porcine blastocysts. (A) Immunofluorescent staining of BrdU in blastocysts from control, DHEA-treated, and IA-treated groups (×400). Scale bar indicates 100 μm. (B) Percentages of proliferating cells in different groups. Values are the mean ± standard deviation the mean. The numbers of embryos examined in each experimental group are shown in the bars. Differences between bars superscripted with different letters (a, b, or c) were statistically significant (p < 0.05).

DNA fragments generated by apoptotic nicking of genomic DNA were measured in individual embryos using the TUNEL assay. The DHEA- and the IA-treated groups displayed significantly higher (p < 0.05) apoptotic cell rates, and significantly lower (p < 0.05) blastocyst cell numbers, compared to the control group (Fig 6A, 6B and 6C). To explore the mechanisms through which DHEA and IA influence the incidence of apoptosis in the parthenogenetic blastocysts, we examined the expression levels of the apoptosis-related genes *Bcl2*, *Bax*, and *Casp3*. Compared to the non-treated group, the levels of *Casp3* and *Bax* mRNA were significantly higher and the levels of *Bcl-2* mRNA significantly lower in the DHEA- and the IA-treated groups (p < 0.05) (Fig 6D). To investigate the reduction in the number of blastocyst cells after exposure to DHEA or IA, we evaluated the proliferative capacity using the BrdU assay. Representative images of cell proliferation in blastocysts are shown in Fig 7A. In both the DHEA- and the IA-treated group, the percentage of proliferating cells was dramatically lower (p < 0.05) than that in the non-treated group (Fig 7B).

## Discussion

PFF derives partly from cumulus cell secretions and partly from follicular vascular exudates. In addition to general nutrients, it contains steroid hormones secreted by the follicular cells, as well as a variety of bioactive substances, and is believed to play important roles in follicle development and maturation [25, 26]. These nutrients and factors safeguard oocyte maturation and promote the completion of meiosis [27, 28]. PFF also protects oocytes from proteolytic attacks, promotes oocyte excretion, and improves successful sperm fertilization chances [29].

In our study, COCs maturated in the presence of 10% PFF, with or without glucose and/or pyruvate supplementation, were able to reach oocyte nuclear maturation and embryo development. However, the presence of 5.6 mM glucose and/or 2 mM pyruvate significantly increased MII-stage oocyte and embryo rates, suggesting that energy metabolism is critical to oocyte maturation and subsequent embryo development. Moreover, the fact that glucose and pyruvate had a similar effect, while combination of the two energy sources did not lead to levels higher than those achieved with single treatments, suggests a correlation between pyruvate and glucose metabolism in the IVM of porcine oocytes. This result is consistent with a previous study, which demonstrated that the addition of exogenous glucose could increase the utilization of pyruvate by mouse COCs and promote meiotic division [1]. Moreover, the fact that 10% PFF was enough to promote nuclear maturation in the absence of glucose and pyruvate supplementation suggests that the amounts of carbohydrates and nutrients in the PFF are sufficient to provide the necessary energy for IVM of oocytes. This strongly indicates that PFF is important to porcine oocyte maturation and succeeding embryo development.

Cumulus cells around the oocyte play a vital role in promoting its maturation [30]. A past study using oocytes stripped of cumulus cells demonstrated that pyruvate was required to promote IVM, whereas adding glucose or lactose did not have a significant effect http://www.sciencedirect.com/science/article/pii/S0093691X07005559—bib53[9]. In contrast, when oocytes were first stripped of cumulus cells and then cocultured with cumulus cells, the addition of glucose was able to promote oocyte IVM [31]. Consistently with these reports, the current study demonstrated that the addition of glucose during IVM of DOs in NCSU37 with 10% PFF was unable to affect maturation and subsequent embryo rates, whereas pyruvate, alone or together with glucose, significantly increased these rates. These results suggest that glucose only can be used by cumulus cells, which mediate its effect on oocyte maturation.

PPP and glycolysis are the two main pathways of glucose metabolism [32]. Thus, both PPP and glycolysis could mediate the effects of glucose [33]. Research on mouse oocytes demonstrated that PPP plays a more important role in promoting maturation than glycolysis [1, 34]. DHEA and IA are widely used inhibitors of PPP and glycolysis, respectively [35, 36]. Addition of DHEA and IA to the IVM medium significantly inhibited oocyte maturation and subsequent development in mice [1, 37], pigs [2] and cattle [38]. Our results were consistent with these previous studies, as addition of DHEA to the IVM medium significantly decreased the MII-stage rate, while both inhibitors significantly reduced the resultant blastocyst rate. We believe that the current study also lays the foundation for further research on the mechanisms through which glucose metabolism regulates porcine oocyte maturation.

In a previous study using already mature porcine oocytes, low levels of intracellular ROS and high levels of intracellular GSH were demonstrated to be important for promoting oocytes maturation and supporting early embryonic development [19, 39]. However, the effects of glucose metabolism on the GSH and ROS content of porcine oocytes are not yet completely understood. In the current study, GSH levels inside cultured porcine oocytes decreased significantly when 200μM DHEA or 2μM IA were added to the normal maturation medium, while DHEA also increased intracellular ROS levels. These results provide further proof that glucose metabolic pathway plays role in maintenance of high level of GSH and low level of ROS.

MPF activity plays a very important role in the cell cycle. Even though previous studies demonstrated that increased ATP content can significantly increase the cellular expression of CDK2, CDK4, Cyclin D, and Cyclin E, they did not report on whether ATP levels affect the activity of MPF in oocytes [40, 41]. In this study, we reduced the amount of ATP in oocytes by suppressing glucose metabolic pathways through treatment with DHEA or IA, and observed that the mRNA levels of the maternal gene *cdc2* as well as the activity of the p34^cdc2^ kinase (which is encoded by *cdc2*) decreased significantly. These results suggest that glucose metabolism might affect the maturation of porcine oocytes by regulating MPF activity.

Changes in the energy supply can cause metabolic reorganization, resulting in an increase in the amounts of ATP and NADPH produced through PPP in mouse [1], porcine [2], and bovine zygotes [42]. PPP may be closely related to the NADPH content of porcine oocytes. In addition, COCs cultured in the presence of both glucose and pyruvate have been shown to give morula with increased developmental capacity [2]. Similar results were obtained in this study; the addition of DHEA or IA significantly reduced oocyte ATP levels, while DHEA also had a significant negative effect on NADH levels. These reductions may mediate the inhibitory effect of the inhibitors on early embryonic development.

It has been reported that high-quality oocytes display decreased levels of *Bax* mRNA, but increased levels of *Bcl-2* mRNA [43]. In this study, we found that DHEA and IA decreased oocyte quality and increased the apoptotic rate in blastocysts; this is consistent with the results of studies in which DHEA was used to promote susceptibility to apoptosis[44]. We also observed that treatment with DHEA or IA resulted in blastocysts with fewer cells, which we attributed to a decrease in proliferation. BrdU analysis showed that DHEA or IA addition to the IVM medium indeed reduced the proliferative capacity of the blastocyst cells, thus our hypothesis was confirmed.

In conclusion, the present study demonstrated that PFF promotes oocytes to make full use of energy. Moreover, glucose plays a key role in IVM of porcine oocytes, promoting cytoplasmic maturation by supplying energy and reducing oxidative stress, possibly through the PPP and glycolysis metabolic pathways.

## Supporting Information

S1 File This is the Raw Data of Figs 1–7. (XLSX)
